# Rare presentation of lues maligna and nodular syphilis in an immunocompetent patient

**DOI:** 10.1016/j.jdcr.2025.09.051

**Published:** 2025-10-27

**Authors:** Mohammed Nasser Al-Abdulla, Aisha AlSrami, Ahmad Hazem Takiddin, Khaled Murshed, Martin Steinhoff

**Affiliations:** a Department of Dermatology and Venereology, Hamad Medical Corporation, Doha, Qatar; b Dermatology Institute, Hamad Medical Corporation, Doha, Qatar; c Translational Research Institute, Academic Health System, Hamad Medical Corporation, Doha, Qatar; d Weill Cornell Medicine-Qatar, School of Medicine, Doha, Qatar; e Department of Laboratory Medicine and Pathology; f Qatar University, Medical School, Doha, Qatar; g Weill Cornell Medicine, New York, New York; h Hamad-bin-Khalifa University, School of Life Sciences, Doha, Qatar

**Keywords:** dermoscopy, histology, HIV, infection, lues maligna, nodular syphilis, therapy

## Introduction

Secondary syphilis can present in many ways; common presentations of secondary syphilis include a generalized papulosquamous or pitriasifrom generalized eruption that commonly affects the palms and soles.^1^ Recently, a rapid rise in the number of syphilis cases globally has been noted, with rare forms of syphilis potentially presenting more frequently.^2^^,^^3^ Lues maligna (LM) is a rare papulonodular or ulcerative subtype of secondary syphilis generally associated with immunosuppression; a recent literature review published in 2021 noted that out of 45 published cases, 33 (73%) were HIV positive.^4^ Here, we present a rare case of a 34-year-old ethnically Arabic, immunocompetent male patient with LM with clinical, dermoscopic, serologic, and histopathologic findings.

## Case report

A 34-year-old male patient with no known past medical history presented to the emergency department with a 3-week history of progressively worsening skin lesions. The lesions initially appeared on his face and gradually spread to involve the trunk and limbs. The patient described the lesions as nonpainful but occasionally itchy. There were no associated oral or mucosal lesions.

In addition to the skin lesions, the patient reported intermittent fever over the past 3 weeks. However, he denied any symptoms related to the upper or lower respiratory tract, gastrointestinal system, or urinary tract. Notably, he had no penile discharge or joint pain.

The patient reported recent travel on an outdoor trip. He mentioned a few mosquito bites during the trip, but denied any other significant exposures and any sexual activity during his trip. He had no known sick contacts, and no family members or friends had similar presentations.

**Fig 1:**
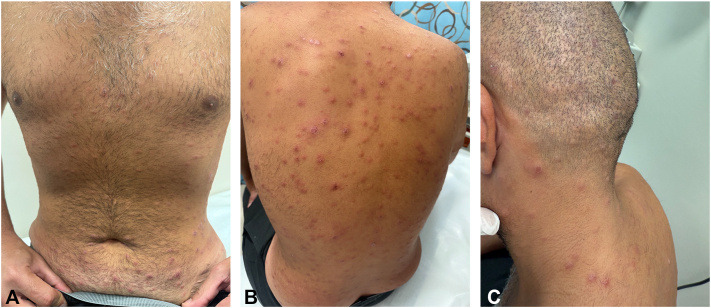
**A-C,** A 34-year-old Arabic man with papulonodular ulcers on the chest, back, and face.

On examination, the patient was vitally stable. On dermatologic examination, the patient presented with widespread erythematous papular and nodular lesions, most concentrated over the back, chest, and face (Fig 1, *A-C*).

**Fig 2:**
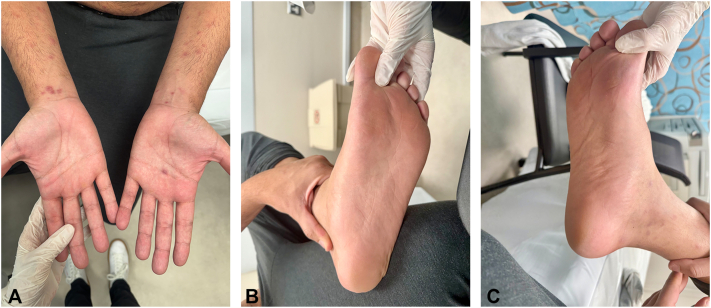
**A-C,** A 34-year-old Arabic man with sparing of the palms and soles apart from a single erythematous scaly papule on the right hand palmar crease.

Notably, there was no oral or mucosal involvement and a single erythematous scaly papule on the right hand palmar crease (Fig 2, *A-C*).

**Fig 3:**
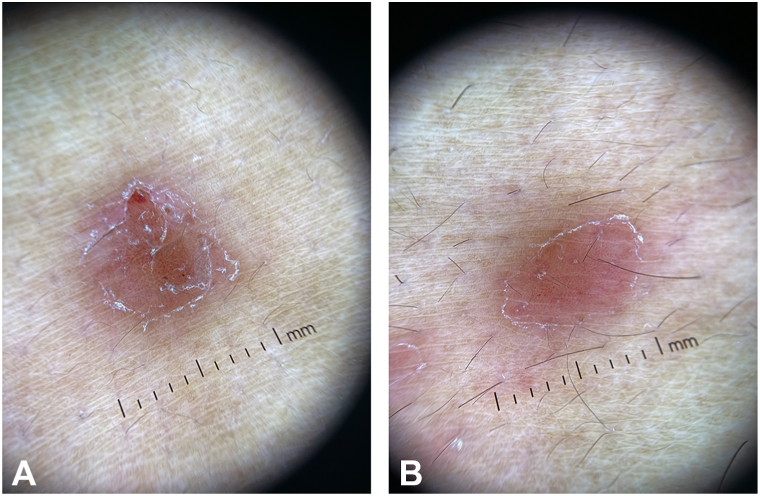
**A,B,** Representative dermoscopic images from papulonodular ulcers on the back, regular pinpoint blood vessels, collarette scales, and some central ulceration.

Dermoscopy revealed regular blood vessels and collarette scales, some lesions with central ulceration (Fig 3, *A, B*).

In addition to the lesions on the trunk, some were also observed on the face and genital area, although these were fewer in number in the latter region. The lesions were a mix of follicular and nonfollicular lesions, further contributing to the varied appearance of the rash. Differential diagnosis included papular urticaria, papular form of pityriasis rosea, and secondary syphilis.

The results of blood tests were unremarkable; however, *Treponema pallidum* antibody was found to be positive with a rapid plasma reagin titer of 1:128, and HIV testing antigen and antibody combo and viral load were negative.

**Fig 4:**
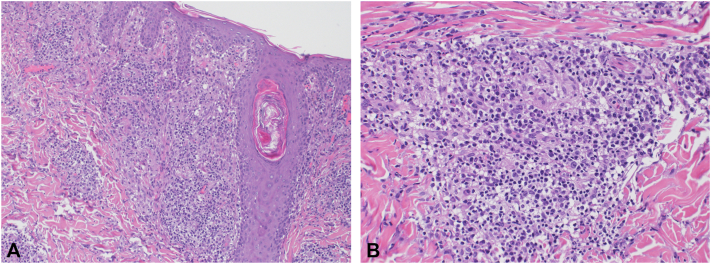
Representative histopathologic images from a 4-mm punch biopsy from the back. **A,** The superficial dermal inflammation is composed of epithelioid histiocytes forming loose granulomas with surrounding lymphocytes and plasma cells **B,** Higher power view showing nonnecrotizing poorly formed granuloma and dense plasma cell infiltrate (**A** and **B,** Hematoxylin-eosin stain; original magnifications: **A,** ×100; **B,** ×200.)

The result of the skin biopsy showed moderate spongiosis and exocytosis. The underlying dermis shows dense perivascular and periadnexal lymphohistiocytic infiltration with focal neutrophils and dense plasma cell infiltrate. There are loose, ill-defined granulomas seen (Fig 4, *A, B*).

Based on the clinical and histopathologic findings, the diagnosis of secondary syphilis was made (LM subtype). The patient received Penicillin G benzathine, 2.4 million units, intramuscular injection weekly for 3 weeks and showed significant improvement with no long-term sequela.

## Discussion

LM is a rare subtype of syphilis, based on a brief literature search that we made using the following terms on PubMed: “malignant syphilis” OR “lues maligna” OR “rupioid syphilis” OR “ulceronodular syphilis.” Only 41 results came back, highlighting the rarity of this presentation. Additionally, the dermoscopic findings of this rare entity are far and few in the literature. Biett sign is a clinical sign that refers to the peripheral scaling around an individual's secondary syphilis rash.^5^ Dermoscopy can further be used to illustrate this finding, in addition to other specific features that may be present.

LM is usually noted in patients with HIV. Our patient denied any past medical history, and his serologic test for both HIV antigen and antibody was negative. He did not have any other history suggesting an immunosuppressed state. He recently started a diabetic medication, tirzepatide, for weight loss 3 months ago and was stopped 2 weeks prior to the visit. This was unlikely to have any effect on the disease presentation because the mechanism of action does not cause any clear immunosuppression. A literature search did not return any association either using PubMed or Google Scholar.

The current rise of syphilis cases worldwide poses a public health issue.^2^^,^^3^ Additionally, it may mean that even rare and uncommon presentations of syphilis may not be as uncommon going forward, such as this case with our patient, who presented with mainly a nodular appearance and some ulceration. This rapid change in numbers will require dermatologists and first-line health care workers to be more aware of rare presentations such as LM and lower their threshold for syphilis, even in cases in which patients are immunocompetent, as highlighted by Wibisono et al,^4^ in which 27% were noted to be immunocompetent. The dermoscopic findings noted combine features of both pityriasis rosea with a collarette scale (Biett sign), in addition to uniform dotted blood vessels that can be seen in psoriasiform lesions. LM may also present with a clear picture of central ulceration; hence, the nodular-ulcerative presentation is more commonly associated with LM. We propose that in the correct clinical setting and when the history is suggestive of syphilis, the aforementioned dermoscopic features, of collaret pitriasifrom scales, regular psoriasiform dotted vessels, and central ulceration, are used as a diagnostic tool to help support the clinical diagnosis of syphilis and, in this case, LM.

## Conflicts of interest

Dr Steinhoff has served on advisory boards for AbbVie, Almirall, Janssen, Sanofi/Regeneron, Novartis, Pfizer, Eli Lilly, Galderma, Leo, BMS, and MenloTx, has been a consultant for AbbVie, Amgen, Galderma, Novartis, Janssen, Pfizer, Eli Lilly, Sanofi, MenloTx, Janssen, Union Tx, Galderma, and Leo, and has received research funding from Galderma, AbbVie, Leo, Pfizer, Novartis, and Sanofi. Drs Al-Abdulla, AlSrami, Takiddin, and Murshed have no conflicts of interest to declare.
